# Survival benefits of oral anticoagulants in patients with pulmonary arterial hypertension: Protocol for a systematic review and meta-analysis

**DOI:** 10.1097/MD.0000000000012203

**Published:** 2018-09-07

**Authors:** Hai-Chao Zhang, Na Wang, Wen Zhang, Zhi-Chun Gu, Xiao-Yan Liu

**Affiliations:** a Department of Pharmacy, Renji Hospital, School of Medicine, Shanghai Jiaotong University, Shanghai; b Department of Pharmacy, The Second Affiliated Hospital of Chongqing Medical University; c Chongqing Medical University, Chongqing, China.

**Keywords:** anticoagulation, meta-analysis, pulmonary hypertension, survival benefits

## Abstract

**Background:**

Pulmonary arterial hypertension (PAH) is an incurable disease with high mortality. Although most studies recommend anticoagulation treatment for idiopathic pulmonary arterial hypertension (IPAH), the survival benefits are uncertain. Therefore, the present paper provides a protocol to investigate this issue by a meta-analysis.

**Methods:**

An electronic search will be performed for randomized controlled trials (RCTs) or cohort studies that reported the interested efficacy data (pulmonary arterial pressure and survival advantage) in anticoagulants-treated patients with PAH. Hazard ratios with their confidence intervals will be calculated using a fixed- or random-effects model.

**Results:**

This study will provide the survival benefits of anticoagulants in PAH patients by pooling the results of individual studies.

**Conclusion:**

The results will bring about vigorous evidence in this issue and guide both clinical decision-making and future research.

## 1 Introduction

In a series of now classical papers published mainly in the 1970s, Wagenvoort et al described the thrombosis of small pulmonary arteries as one of the typical histopathological features in idiopathic pulmonary arterial hypertension (IPAH), so-called primary pulmonary hypertension at that time.^[1–3]^ This observation, together with studies demonstrating the hypercoagulability in patients with severe pulmonary hypertension (PH), led to the hypothesis that in situ thrombosis of altered pulmonary vessels may contribute to disease progression.^[4–6]^ Thus, therapeutic anticoagulation might provide certain beneficial effects. For supporting this issue, Fuster et al carried out an observational study that confirmed a better survival with anticoagulants treatment.^[7]^ Similarly, a later study by Rich et al also showed the improved survival in patients receiving anticoagulants.^[8]^ However, several studies recently showed a negative result on patients with IPAH or other types of pulmonary arterial hypertension (PAH).^[9–11]^ In general, no randomized controlled trials (RCTs) and high-quality cohort studies has focused this fields. For a robust result, we therefore aim to summarize current available evidences regarding the association between the use of anticoagulants and survival benefits in PAH.

## 2 Methods

### 2.1 Data sources and searches

This study will be conducted in accordance with PRISMA Statement and is registered in PROSPERO (registration number: CRD42018103863). Electronic database of Medline, Embase, and Cochrane library will be searched using MeSH words and free words with the following searching strategy: “pulmonary hypertension” or “pulmonary arterial hypertension” or “PAH” in combination with “anticoagulant.” Additionally, unpublished trials will be identified from the ClinicalTrials.gov Website. References of all pertinent articles will also be scrutinized to ensure that all relevant studies are identified.

### 2.2 Study selection and eligibility criteria

The following criteria will be used: studies design must be RCTs or observational studies; the patients should be PAH and received anticoagulation treatment or not between 2 groups. The primacy outcome of survival effects is reported in 2 groups, respectively.

### 2.3 Data extraction

For each study, the following data will be extracted: general data (study design, year of publication), population characteristics (numbers, mean age, sex, and mean pulmonary arterial pressure), and treatment (anticoagulation use). Data of survival effects will be collected in 2 treatment groups.

### 2.4 Quality evaluation

The methodological quality of cohort study will be evaluated using the Newcastle-Ottawa Scale (NOS) criteria.^[9]^ The NOS criteria is based on three categories: subject selection (representativeness of the exposed cohort, selection of the non-exposed cohort, ascertainment of exposure, and demonstration that the outcome of interest was not present at the start of the study); comparability of subject (comparability of cohort on the basis of the design or analysis); and clinical outcome (assessment of outcome, follow-up long enough for outcome to occur, and adequacy of follow-up of the cohort). NOS scores range from 0 to 9 with scores ≥7 indicating good quality, and poor to moderate quality when the score was <7.^[12]^ For each study, we will also assess how the population is selected, the duration and route of medication administration, and the funding source.

### 2.5 Bias assessment

Potential publication bias will be assessed by visually inspecting funnel plot and will be minor if the plot of the magnitude of treatment effect in each study versus its precision estimate showed an approximate symmetrical funnel shape.^[13]^

### 2.6 Data analysis

Statistical analyses will be performed using the R statistical software with meta packet (version 3.4.4). Individual studies and meta-analysis estimates will be derived and presented in forest plots. Results will be reported as hazard ratios with their 95% confidence intervals. Heterogeneity will be evaluated using the *I*^2^ test that measures the percentage of total variation between studies.^[14]^ For the meta-analysis, a fixed-effects model will be applied if appropriate levels of heterogeneity are found to be present. While if moderate heterogeneity levels exist, a random-effects model will be applied using the Mantel–Haenszel method.^[15]^

## 3 Discussion

PAH is an incurable disease with high mortality. Although most studies recommend anticoagulation treatment for IPAH, the benefits are uncertain. And there are few studies that confirm the positive effects of anticoagulation treatment for other types of PAH. The present study will focus this issue by pooling current evidences and will provide both clinical decision-making and future research. Several possible limitations might be addressed in our study. Firstly, we recognize an inherent property of non-RCTs, which may introduce certain bias when regarding population selection and confounding factors. However, all the included studies might be retrospective study as no RCTs reported in this field. Secondly, the anticoagulant treatment in most of the studies is warfarin and no novel oral anticoagulants or heparin is available. Thirdly, most studies will not classify the PAH explicitly, making the limitation of corresponding sub-group analysis. Finally, none studies are involved in target INR value, which may lead to the difference of anticoagulant effects among studies. In Europe, the target INR for PAH anticoagulation is 2.0 to 3.0 and temporary interruptions are usually bridged with heparin or analogues.^[16]^ In contrast, the US PAH centers target an INR of 1.5 to 2.5 for PAH patients with no bridging for temporary interruptions.^[17]^

## Author contributions

Hai-Chao Zhang and Zhi-Chun Gu conceived the idea and design for this systematic review. Hai-Chao Zhang, Na Wang, and Wen Zhang developed the methodology for the systematic review protocol. The contents of this manuscript were drafted by Hai-Chao Zhang with input from all members of the authorship team. The manuscript was reviewed by Zhi-Chun Gu and Xiao-Yan Liu for important intellectual content. All authors read and approved the final manuscript.

**Conceptualization:** Hai-Chao Zhang, Na Wang, Wen Zhang, Zhichun Gu, Xiao-Yan Liu.
